# Customized Additive Manufacturing in Bone Scaffolds—The Gateway to Precise Bone Defect Treatment

**DOI:** 10.34133/research.0239

**Published:** 2023-10-09

**Authors:** Juncen Zhou, Carmine Wang See, Sai Sreenivasamurthy, Donghui Zhu

**Affiliations:** Department of Biomedical Engineering, Stony Brook University, Stony Brook, NY, USA.

## Abstract

In the advancing landscape of technology and novel material development, additive manufacturing (AM) is steadily making strides within the biomedical sector. Moving away from traditional, one-size-fits-all implant solutions, the advent of AM technology allows for patient-specific scaffolds that could improve integration and enhance wound healing. These scaffolds, meticulously designed with a myriad of geometries, mechanical properties, and biological responses, are made possible through the vast selection of materials and fabrication methods at our disposal. Recognizing the importance of precision in the treatment of bone defects, which display variability from macroscopic to microscopic scales in each case, a tailored treatment strategy is required. A patient-specific AM bone scaffold perfectly addresses this necessity. This review elucidates the pivotal role that customized AM bone scaffolds play in bone defect treatment, while offering comprehensive guidelines for their customization. This includes aspects such as bone defect imaging, material selection, topography design, and fabrication methodology. Additionally, we propose a cooperative model involving the patient, clinician, and engineer, thereby underscoring the interdisciplinary approach necessary for the effective design and clinical application of these customized AM bone scaffolds. This collaboration promises to usher in a new era of bioactive medical materials, responsive to individualized needs and capable of pushing boundaries in personalized medicine beyond those set by traditional medical materials.

## Introduction

Orthopedic implants serve 3 primary purposes: fixation, replacement, or regeneration. Fixation implants—such as pins, screws, nails, and plates—are used to stabilize fractured bones, typically allowing for self-healing by the host tissue. Depending on the clinical circumstances, these fixation implants might be removed or permanently left in place.

In instances where self-healing is unattainable, due to severe trauma or other pathologies, the implant assumes the role of a replacement for the damaged bone. Commonly, these replacement implants are nonbiodegradable and remain within the patient's body for a lifetime. Examples include prosthetic implants used in hip replacement surgeries and reconstructive jaw implants. However, in certain scenarios, even when bone defects are critically sized, bone regeneration within the defect region is achievable with the aid of bioactive bone implants.

The gold-standard treatment for bone defects has traditionally been autologous bone grafts. However, this method has limited availability and is associated with high clinical risks due to the surgical procedures involved. An alloplastic graft, with appropriate mechanical properties and superior osteocompatibility, could serve as an ideal strategy for clinicians [1]. Yet, several challenges persist in clinical scenarios, especially considering the complex structure of bone tissue [2]. Consequently, the design of a bone scaffold must consider its intricate architecture, posing major challenges to the fabrication process. Furthermore, bone defects—caused by trauma, tumors, or infections—can vary in location, shape, and dimensions. This variance necessitates a precision medicine approach, where bone scaffolds are tailored to the specific needs of each patient.

Additive manufacturing (AM), also known as 3-dimensional (3D) printing, emerges as a compelling solution. It is a layer-by-layer fabrication method capable of rapidly creating complex structures using computer-aided design (CAD). By utilizing medical imaging data, AM can produce bone scaffolds with high precision and intricate design. The entire fabrication process can be customized according to the patient's needs, including the scaffold's contour profile, porous structure design, material selection, and posttreatment. Thus, the features of AM make it a robust tool for providing precision treatment for bone defects.

This review focuses on the pivotal role of customization in bone scaffold fabrication and the treatment of bone defects. First, we elucidate the necessity of customized AM bone scaffolds in treating bone defects. We then present a comprehensive overview of the AM bone scaffold fabrication process. This process commences with the diagnosis and imaging of bone defects, which guide clinicians and engineers in designing scaffolds with suitable materials and geometry. Following the design phase, the design is then transformed into a scaffold through a fast, precise, and flexible approach. The review delves into the customization at each step of the scaffold fabrication process and concludes with a discussion on future directions in customized AM bone scaffolds, including a model for clinical cooperation between clinicians and engineers.

Customization stands as both the solution and challenge in bone scaffold applications. AM, however, may be the most effective approach to meet this requirement. Through this review, our aim is to equip clinicians and engineers with an exhaustive understanding of customized AM bone scaffolds and to promote advancements in this field beyond theoretical discussion.

## The Current State of the Art in AM Scaffolds for Bone Defect Treatment

### Two treatment purposes of customized AM scaffolds: Bone regeneration and reconstruction

**Fig. 1.**
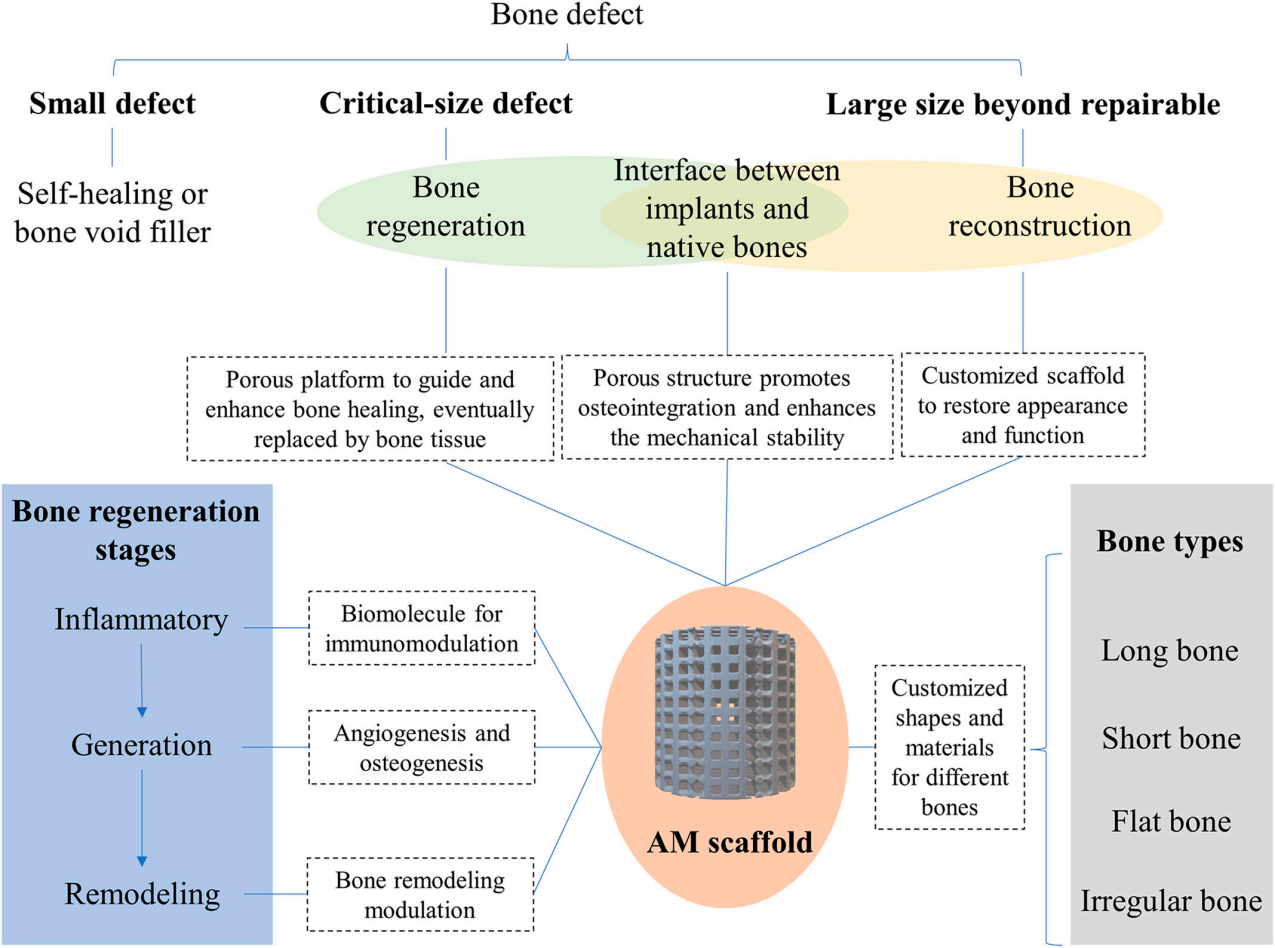
The correlation between the customized AM scaffold and the treatment of bone defects.

When a bone defect requires intervention, the focus of implants is either regeneration, restoring the bone's biological and mechanical functions, or reconstruction, preserving appearance or functionality. Factors such as age, gender, and health conditions greatly influence bone quality and dimensions, which is especially important considering that most bone defects are consequences of trauma or tumors. This results in considerable variation in the geometry of defects on a case-by-case basis. Unlike conventional implants with standardized sizes and shapes, custom AM bone scaffolds can be tailored to align precisely with specific defects, utilizing information gathered during defect assessment. The relationship between custom-designed AM scaffolds and bone defects is illustrated in Fig. 1.

#### The influence of customized AM scaffolds in different stages of bone regeneration

Bone defects, generally caused by trauma injuries or pathological conditions like osteoporosis, osteopenia, and bone cancer, often necessitate implant interventions. Natural bone healing cannot always suffice for major defects, hence the need for scaffolds. Implants must integrate effectively with the existing bone to aid regeneration, a complex, long-term process. This process involves (a) an inflammatory stage (hematoma formation), (b) bone generation (characterized by the appearance of fibrocartilaginous callus, bony callus, and revascularization), and (c) bone remodeling, where the bony callus is remodeled by osteoblasts and osteoclasts [3,4]. Customized AM bone scaffolds offer specific advantages at each of these stages of bone regeneration.

1. Inflammatory stage

Upon applying an implant to a bone defect, various inflammatory reactions are triggered. The behavior of these reactions can be steered by customized AM bone scaffolds. A widely used modulation strategy involves directing macrophage polarization toward the M2 phenotype as opposed to M1. Scaffolds composed of diverse materials, 3D-printed, have demonstrated success in steering macrophage polarization. These include polylactic-co-glycolic (PLGA) scaffolds modified by human umbilical cord mesenchymal stem cells (MSCs)-derived extracellular matrix (ECM) [5], nanoscale bioactive glass scaffolds [6], and multicell-laden scaffolds that incorporate bone morphogenic protein-4 [7]. These scaffolds have illustrated their ability to stimulate M2 macrophage polarization and to promote healing of bone defects. The proinflammatory tumor necrosis factor-α, which can obstruct bone regeneration when present at high levels, has been a subject of study. Scaffolds of 3D-printed Atsttrin-incorporated alginate/hydroxyapatite (HAP) demonstrated suppressive effects on tumor necrosis factor-α and enhanced bone defect repair [8]. Additionally, 3D-printed poly(propylene fumarate) scaffolds produced desirable inflammation scores, and β-tricalcium phosphate (β-TCP) scaffolds effectively curbed the expression of genes related to inflammation [9].

2. Bone generation stage

During the bone generation stage of healing, 3D-printed scaffolds can enhance both angiogenesis and osteogenesis. For instance, 3D-printed titanium scaffolds have been shown to promote collagen mineralization while also boosting angiogenesis and osteogenesis in situ [10]. Similar effects have been observed with 3D-printed ceramic scaffolds [11]. Compared to unmodified β-TCP scaffolds, those integrated with mesoporous bioactive glass have demonstrated superior responses in osteogenesis and angiogenesis [12]. A separate study, which infused MgO and SiO_2_ into β-TCP scaffolds, found that the subsequent release of Mg^2+^ and Si^4+^ stimulated the formation of both bone and blood vessels [13]. Moreover, the role of pore architecture in calcium-deficient HAP scaffolds on osteogenesis has been explored [14].

3. Bone remodeling stage

During the final phase of bone healing, bone remodeling directed by osteoblasts and osteoclasts reshapes the newly formed bone according to the original structure and mechanical load. Few studies have explored bone remodeling in the context of 3D-printed scaffold incorporation. One such study employed scaffolds composed of medical-grade polycaprolactone (PCL) and β-TCP to investigate the biomineralization process at the soft-to-bone interface [15]. Findings indicated that basic multicellular units remodeled bone near the native cortical bone. Another investigation used a hybrid scaffold comprising PLGA/TCP/icariin in a rabbit model, monitoring dynamic bone remodeling in tandem with scaffold degradation [16]. Further research involving a 3D-printed PCL scaffold integrated with aspirin liposomes and bone forming peptide-1 highlighted the scaffold's potential to promote bone remodeling, primarily via the phosphoinositide 3-kinase/protein kinase B (PI3K/Akt) signaling pathway [17].

#### Customized AM scaffolds in bone reconstruction

In some instances, the priority leans toward bone reconstruction over regeneration, particularly for bones connected to physical appearance, like cranial and maxillofacial bones, or those adjacent to joints, such as the humerus and acetabular bones. Customized 3D-printed scaffolds can effectively restore the original bone's appearance and function, with their porous structures promoting integration with native bone. Clinically, 3D-printing techniques have proven useful for large-area bone defects, with applications in cranioplasty for prosthesis molds or skull implants [18–21]. For areas close to joints, 3D-printed scaffolds have been utilized for substantial segmental bone defects in the tibia [22], acetabular revision surgeries [23], and severe humerus defects where traditional prostheses are unsuitable [24].

### Clinical and preclinical trials of customized AM scaffolds applied for different types of bones

**Fig. 2.**
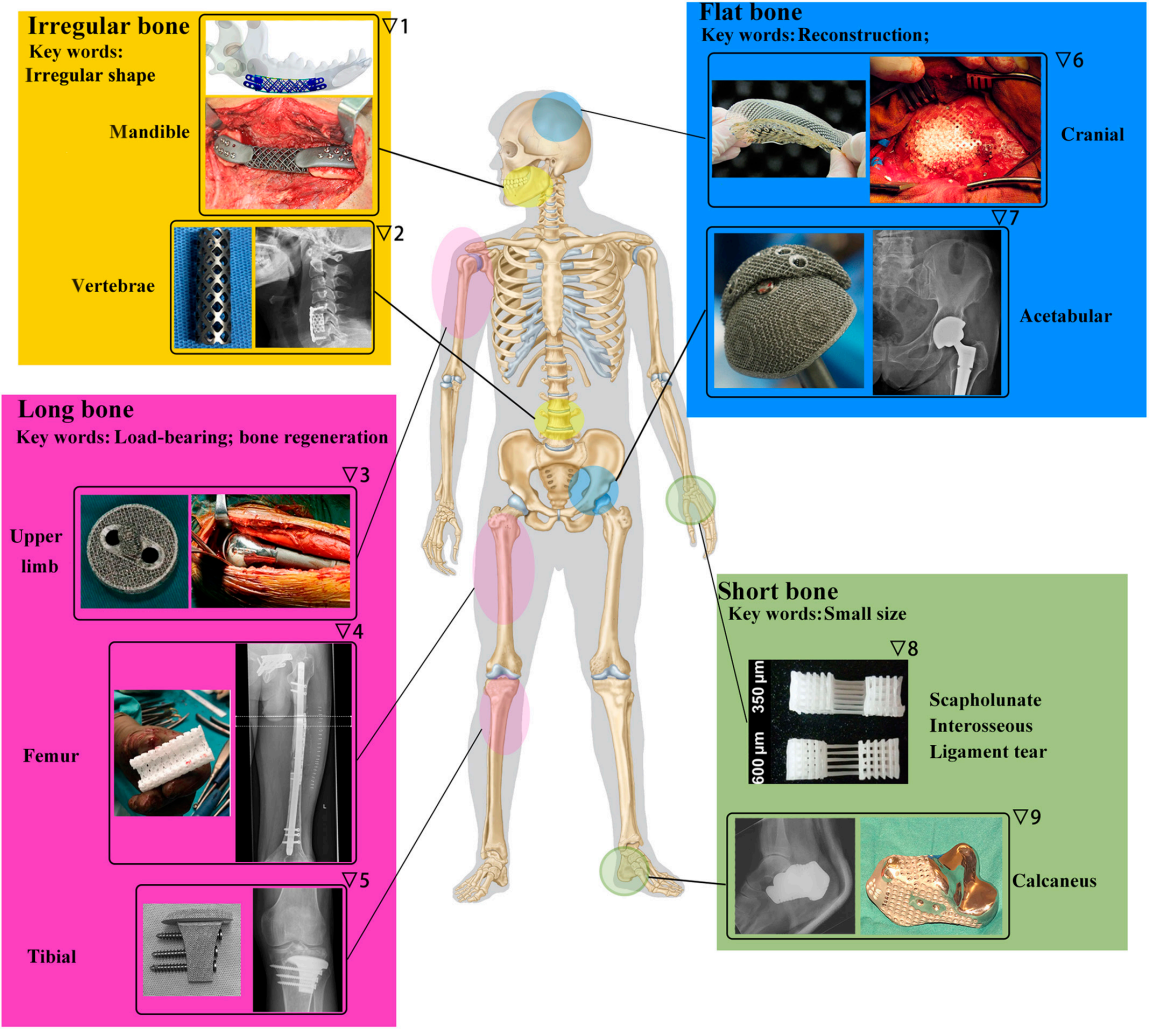
The clinical applications/studies of customized AM scaffolds on different types of bones. Pictures are adapted with permission from refs. ▽1 [253], ▽2 [254], ▽3 [255], ▽4 [31], ▽5 [36], ▽6 [256], ▽7 [23], ▽8 [41], and ▽9 [257].

Bones can generally be categorized into 4 types: (a) long bones, (b) short bones, (c) flat bones, and (d) irregular bones. The nature of defects that occur in these different bone types exhibit distinct characteristics, and therefore, the corresponding AM bone scaffolds tailored for these situations possess unique structures and properties. Figure 2 showcases clinical/preclinical trials of custom-made AM scaffolds applied to various bone types.

**Fig. 3.**
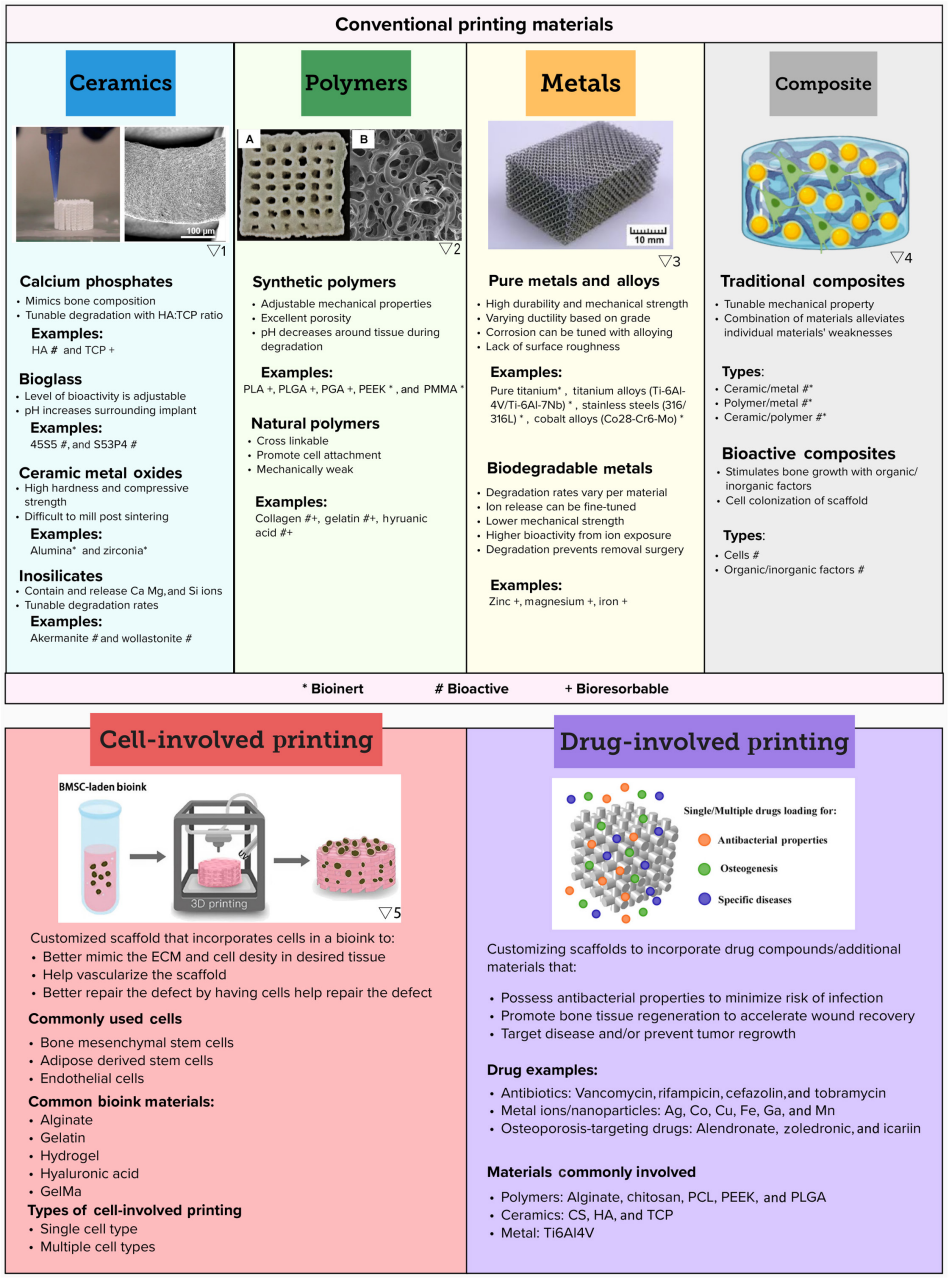
The selection panel of materials for customized AM scaffolds. (A) Application scenarios. (B) Properties of different types of materials and their applicable scenarios. Pictures are adapted with permission from refs. ▽1 [258], ▽2 [259], ▽3 [260], ▽4 [261], and ▽5[262]. PGA, poly(glycolic acid).

#### Long bone cases

Long bones are composed of 2 types of tissue—trabecular (spongy) and cortical (compact)—that surround the central bone marrow. The function a bone serves primarily determines the cortical-to-trabecular tissue ratio. Cortical bone, made up of osteon cylinders, is essential for resisting bending, while trabecular bone resists compression. Key elements within these structures include osteocytes and Haversian canals housing blood vessels and nerves [25,26]. When long bone segmental defects occur, requiring scaffolds for repair, these scaffolds must exhibit advanced biological, mechanical, biodegradable, and architectural properties. The architecture is vital, as scaffolds should have porous, interconnected structures that support and induce defect regeneration. Given the need for patient-specific optimization of architecture and properties like porosity, AM is well suited for this kind of bespoke fabrication.

Upper limb humerus defects often result from injuries or bone tumors and can be involved in surgeries like total shoulder arthroplasty. In one instance, a chondrosarcoma patient with a loose proximal humeral replacement prosthesis required a custom 3D-printed titanium alloy shoulder prosthesis, fabricated using electron beam melting (EBM) with a 60% porosity [24]. After 1 year, the prosthesis remained stable and functional. Separately, 3D-printed titanium-mesh scaffolds with varied stiffness were tested on a critical humerus model in sheep, with lower stress shielding promoting early bridging and increased endochondral bone formation [27]. Another study examined the biological fixation between tendon and prosthesis, using selective laser melting (SLM)-fabricated Ti-6Al-4V scaffolds with different pore sizes implanted into rabbit humerus heads [28]. A size of 527.15 μm was found optimal for tendon growth. Two further studies explored in situ bone repair aided by 3D printing in humerus defect models [29,30].

Long bones in the leg, particularly the femur and tibia, are frequently researched in the context of 3D-printed scaffolds. For a patient with a femur shaft fracture, a patient-specific 3D-printed hybrid scaffold, composed of medical-grade PCL and tricalcium phosphate (TCP), was successfully used, resulting in bony fusion and interconnection after a year [31]. In another study, 5 patients with femur defects used customized 3D-printed micro-porous prostheses, resulting in rapid weight-bearing and good hip and knee joint scores [32]. Additional studies explored other aspects of 3D-printed scaffolds, such as bacterial infection inhibition, vascular ingrowth, and regional gene therapy [33–35].

In the case of tibia defects, 3D-printed scaffolds have shown promising results. For example, one patient, following the removal of giant cell tumors, benefitted from a combination of an autograft and a 3D-printed porous implant, achieving satisfactory limb function after 29 months [36]. Another patient, with a tibia fracture, experienced no functional limitations after a custom 3D-printed titanium scaffold was applied [22]. Studies also explored different materials for 3D-printed scaffolds, including β-TCP, which was shown to promote directional regeneration and remodeling of bone defects [37]. Further research discovered that a scaffold with a controlled pore size of 400 μm offered the best bone formation capacity [38]. Moreover, 3D-printed polymer scaffolds, enhanced with compounds and cells like alendronate, recombinant human bone morphogenetic protein-2, and MSCs, have been tested for their potential to optimize bone regeneration [39,40].

#### Short bone cases

Short bones, such as carpal and tarsal bones, primarily consist of spongy bone encased in a compact bone layer, distinguished by their small size and cubic shape. For frequent wrist injuries like scapholunate interosseous ligament tears, multiphasic bone-ligament-bone scaffolds, fabricated through AM, emulate native tissue structure and facilitate bone formation when combined with MSCs [41].

The calcaneus, a tarsal bone bearing most of the foot's weight, has been remediated for tumor-induced defects using a personalized 3D-printed solid Ti-based alloy implant, augmented with a mesh for ligament suturing. This method preserved the Achilles insertion site and enabled unaided walking [42]. Another remedy for calcaneal defects, the Masquelet technique, combines 3D printing with the induced membrane technique. In one study, calcaneal defects were reconstructed using 3D-printed custom molds for shaping bone cement, resulting in a highly matched repair [43].

#### Flat bone cases

Flat bones, like short bones, contain spongy bone sandwiched between 2 compact layers. Yet, as broad plates, they serve as shields for underlying tissue and attachment points for muscles. Acetabular and cranial bones, among flat bones, frequently suffer defects from trauma, cancer, and other ailments.

In hip revision surgeries, highly porous trabecular titanium cups, produced by EBM, have been utilized for acetabular revisions [23,44]. This approach has demonstrated positive midterm outcomes, including early patient mobilization, weight bearing, and no modularity failures [23]. For complex acetabular bone defects—common in primary and revised total hip arthroplasty—3D-printed porous augments have proven beneficial. In a swine model, these augments, created using Ti6Al4V powders, demonstrated desired biomechanical properties and tissue compatibility [45].

Cranioplasty, or skull bone reconstruction, seeks to restore natural contours. The process, however, is complicated by the curved skull bone surface, complex maxillofacial geometries, and size of the bone defect, which varies depending on the patient's condition.

Several scaffolds have been devised to treat cranial defects. Polymethylmethacrylate (PMMA) and polyetheretherketone (PEEK) implants have been clinically used to address these defects, aided by 3D-printing techniques to create patient-custom molds [46–50].

An alternative approach uses the implant materials directly in 3D printing based on 3D-computed tomography (3D-CT) data. Titanium (Ti)-based meshes have been used for this purpose given their chemical and biological stability. These meshes have been successfully applied in cranioplasty for patients with large skull defects and recurrent infections [20]. A comparative study found no significant differences in postoperative complications between 3D-printed Ti mesh and autologous bone flap, suggesting that material selection should be patient-specific [21]. Interestingly, combining 3D-printed Ti porous implants with calcium phosphate fillers showed promising results, potentially lowering failure rates, reducing surgery times, and increasing infection resistance [51,52]. Furthermore, 3D-printed cranioplasty scaffolds may prove more cost-effective than traditional implant methods [53,54].

#### Irregular bone cases

Irregular bones like the mandible (lower jaw) and vertebrae, consisting of spongy bones enclosed within compact bone, often necessitate implants due to trauma, disease, and tissue degeneration.

A patient-customized mandibular prosthesis, made of Ti6Al4V, was fabricated using EBM to successfully reconstruct a mandibular defect. The prosthesis incorporated porous structures at the upper and lower ends and showed no complications over a 9-month follow-up period [55]. In a rabbit mandibular defect model, magnesium-substituted wollastonite and β-TCP scaffolds demonstrated good osteogenic capability [56,57]. Poly(lactic acid) (PLA)/HAP/β-TCP and PCL/β-TCP scaffolds were used to reconstruct mandibular defects in dog models, while PLGA/HAP scaffolds were applied to rat mandibular bone defects, all showing promising results for mandibular reconstruction [58–60]. Natural polymers such as alginate, chitosan, and gelatin have also been considered for scaffolding materials. The alginate/TCP/HAP scaffold provided not only structural stability and in vitro cytocompatibility but also antibacterial function [61]. Similarly, nano-HAP (nHAP)/chitosan/gelatin scaffolds promoted mandibular bone regeneration in a swine model [62].

Spinal fusion, a surgical technique joining adjacent vertebrae with bone grafts, is commonly used for spinal disorders. A study used a 3D-printed scaffold made of PLGA, HAP, and human demineralized bone matrix in a rat posterolateral spinal fusion model [63]. This scaffold had impressive fusion scores and increased osteogenesis-associated genes expression, revealing the impact of scaffold geometry and architecture. The optimal configuration for osteointegration and fusion appeared to be larger pore size and aligned struts at a 45° angle [63]. In another study, PLGA/β-TCP composite scaffolds with salvianolic acid B improved bony fusion by enhancing osteogenesis and angiogenesis [64]. Beyond polymer–ceramic combinations, research has also explored metallic biomaterials. For instance, 3D-printed interconnected titanium alloy scaffolds filled with HAP, implanted in a sheep model, showed superior osteogenic performance compared to scaffolds without HAP [65].

## The Fabrication Flow of Customized AM Bone Scaffolds

While AM bone scaffolds demonstrate considerable advantages in treating bone defects, their fabrication process is complex, is multidisciplinary, and requires extensive collaboration. Each scaffold must be tailored to the patient's needs, making customization crucial throughout the process. This review will explore 4 key aspects of design and fabrication: bone defect imaging, material selection, scaffold design, and fabrication method. We will delve into the importance of customization in each of these stages.

### Bone defect imaging for customization

The initial step in fabricating a customized scaffold involves obtaining precise imaging data of the patient's bone defect. To ensure optimal integration at the tissue-scaffold interface, accommodate mechanical adaptations, and facilitate other personalized scaffold functions, highly accurate imaging is essential. This is especially critical for defects resulting from tumors or trauma, typically characterized by irregular shapes. Through detailed medical images, clinicians and engineers can evaluate the bone defect's anatomy and develop a scaffold design that satisfies the specific requirements.

#### General imaging process

CT and magnetic resonance imaging (MRI) are primarily utilized for acquiring 3D imaging data, given their ability to capture isotropic or near-isotropic datasets [66]. While MRI offers the advantages of zero radiation exposure risk and precise delineation of soft tissue anatomy, its efficacy in capturing thin z-section slices can be compromised by movement-induced artifacts [67,68].

In the orthopedic domain, CT images, known for their high contrast, serve as the main data source for image postprocessing, often supplemented by MRI. Modern multirow detector computer tomography, a CT technique variant, can capture thin-section slices less than 1 mm thick, enhancing 3D printing image postprocessing [69–71]. cone beam CT proves particularly useful for imaging oral and maxillofacial areas [72], offering reduced radiation exposure and accurate 3D volumetric data across axial, sagittal, and coronal planes [73]. Regardless of the imaging method, the resultant images are stored as Digital Imaging and Communications in Medicine files for subsequent processing.

Digital Imaging and Communications in Medicine files are processed using image postprocessing software to extract 3D projects from segmented regions of interest, with the thresholding voxel intensity value identifying the bone tissue. These projects' contours are then transformed by CAD software, and the CAD data is stored as Stereo Lithography (STL), a standard 3D file format [74]. Engineers can then alter the acquired STL file to fabricate a customized 3D-printed scaffold with 3D printers.

#### Imaging of orthopedic hardware

Orthopedic hardware, particularly metallic implants, can degrade the quality of CT and MRI images. Nevertheless, imaging such hardware can be crucial in certain cases such as replacing dysfunctional implants, where imaging provides vital insights into defect anatomy, scaffold design, and surgical planning. Postsurgical imaging is also crucial for evaluating implant positioning and stability, assisting clinicians in determining the need for further revisions.

CT image acquisition is affected by metallic hardware due to beam hardening, scatter effects, splay artifacts, and nonlinear partial volume effects. The implants' alloy compositions, dimensions, and geometry can also impact CT image artifacts [75]. Various strategies can minimize these effects, including modifying tube voltage and current to reduce beam hardening, though this needs to be balanced against patient dose limitation and decreased soft tissue contrast [76]. Scatter artifacts can be minimized with an antiscatter grid, while splay artifacts can be mitigated using a z-flying focal spot [77]. Additional solutions include altering CT reconstruction filters and algorithms [78,79], and employing dual-energy techniques [80].

In the case of MRI, metal implants can induce an electrical current, generating magnetic distortion and image artifacts. The extent of these artifacts is influenced by the implant's susceptibility [75]. Techniques to reduce metal artifacts include using lower static field strength [81], increasing receiver bandwidth [82], and utilizing metal artifact reduction sequence techniques, such as view angle tilting, multiacquisition variable-resonance image combination, and slice encoding for metal artifact correction [83,84].

### Materials selection for customized AM bone scaffolds

Biomaterials for bone scaffolds should exhibit biological properties (such as biocompatibility, osteoinductivity, and osteoconductivity), mechanical strength, and processability. These materials can be broadly categorized into ceramics, polymers, metals, and composites. Furthermore, the incorporation of cells and drugs in the printing materials can impart specific functionalities, as depicted in Fig. 3. The optimal choice of materials for custom scaffolds relies on several factors: the location and physiological properties of the bone defect, the scaffold's purpose (such as aesthetic or mechanical support), its expected lifespan and biodegradation rate, and additional custom functions like antibacterial action or osteogenesis enhancement. The upcoming sections will briefly review the materials used in customized scaffolds.

#### Ceramics

Ceramics utilized in AM can be categorized into 2 types: bioactive and bioinert ceramics. These materials generally exhibit excellent biocompatibility, and their elemental compositions can be tailored to achieve specific in vivo properties. Despite offering commendable compressive strength and corrosion resistance, their inherent brittleness leads to lower fracture strength in comparison to other materials [85].

1. Bioactive ceramics

TCP [86,87], calcium sulfate (CS) [88,89], HAP [90,91], akermanite [92], diopside [93,94], and bioglass [95] can be classified into bioactive ceramics, which generally are biodegradable and can induce osteoconductivity and osteoinductivity.

TCP and CS are bioresorbable materials extensively used in treating bone defects, particularly for load-bearing structures, due to their resorption rates aligning well with bone tissue regeneration. Both materials come in 2 forms, α and β, which degrade at different rates, faster than HAP. While β-TCP is often combined with HAP to form biphasic TCP, enabling controlled degradation rates, α-TCP's lower density and stability make it more suitable for cement-based applications [96,97]. The combination of CS with β-TCP or HAP in bone constructs has been shown to enhance stability and pressure resistance, due to the formation of calcium-deficient HAP and dihydrate CS crystal lattices. The beneficial outcomes of these combinations have been widely explored in the context of healing osteogenic defects [98]. Investigations into different ratios of CS/HAP and CS/β-TCP powders in printed scaffolds have indicated that a 25:75-wt.% coarse HAP:CS powder mixture offers superior compressive strength, wettability, and ideal pore diameters for cell attachment. Additionally, the incorporation of dopants, such as metal ions or compounds, into ceramic materials can enhance physical and biological properties, including osteoinduction [99]. Particularly, studies on Fe-Si doped β-TCP scaffolds have revealed an increased mechanical strength, enhanced mineralization, and angiogenesis compared to pure β-TCP scaffolds. Similar results have been obtained with β-TCP scaffolds doped with SiO_2_ and ZnO, achieving desirable degradation rates, improved densification, long-term stability, and interconnected osseous tissue [100,101]. Further research on the surface geometries or porosities of printed TCP and CS-based scaffolds has shown promising biocompatibility and osteogenic capabilities [87,89,102].

HAP, a bioactive ceramic naturally present in bone tissue, has been used in AM to create bone scaffolds. Recent research shows that pure HAP scaffolds exhibit good biocompatibility and can mimic the interconnected pore structures and topography of the native bone matrix [91,103]. In one study, HAP scaffolds were prepared using digital light processing (DLP) with 20-nm HAP powder and photopolymer, resulting in postsintered scaffolds that promoted the proliferation and attachment of MC3T3-1 cells. However, HAP's limitations, including its brittleness, poor degradation capacity, and limited resorption, may hinder its clinical applications [104]. These issues could potentially increase fracture risks around implant sites and necessitate permanent fixtures rather than facilitating complete replacement of new bone tissue [97]. Nevertheless, these challenges can be mitigated by incorporating HAP into composite materials.

Inosilicate materials such as akermanite, diopside, and wollastonite show high bioactivity due to the release of Ca, Mg, or Si ions, promoting mineralization, osteogenesis, and angiogenesis. Akermanite cages, printed using direct-ink writing and enhanced with 15% or 30% bioglass, show increased compressive strength, a key to lowering melting temperatures and reducing pressurization and sintering needs for maintaining porous structure. The enhanced cages exhibited enhanced angiogenic stimulation, promoting bone regeneration and maintaining long-term stability and osteogenic adhesion in spinal fusion areas [92,105]. Similarly, diopside combined with bioglass has shown improved compressive strength and angiogenic capabilities in orbital implants. Separate research used varying diopside and wollastonite ratios to produce high-resolution, sturdy, high-porosity diamond lattice scaffolds via digital light printing, demonstrating a promising direction for future applications [93,94].

Bioactive glasses, ceramic mixtures composed of body-native materials including SiO_2_, CaO, Na_2_O, and P_2_O_5_, are excellent for bioceramic scaffold materials. Their dense, negatively charged surface encourages serum protein absorption, and the raised pH around implants offers antimicrobial properties [106,107]. Two percent Fe_2_O_3_ doped bioglass has been used for creating alveolar bone substitute scaffolds, demonstrating good biocompatibility and upregulation of osteogenic markers [108].

With their osteogenesis-promoting and degradation abilities, bioactive ceramics are ideal for bone regeneration scaffolds. These constructs stimulate natural bone healing, eventually restoring movement and load-bearing capabilities. By altering material properties, the degradation rates and mechanical properties can be tailored to specific needs, making them suitable for various applications, both load-bearing and non-load-bearing.

2. Bioinert ceramics

Bioinert ceramics like alumina and zirconia are recognized materials for implant fabrication in AM. Alumina, one of the earliest bioceramics used clinically, offers low wear, high stability, and compressive strength. Studies show that patterned, micropillar alumina surfaces can promote osteogenic behaviors in human mesenchymal stem cells [109]. Although alumina's strong mechanical properties suit load-bearing applications, its brittleness restricts use in fracture scenarios. It requires bioactive substrate coatings or biomolecules to improve integration with the surrounding environment due to its bioinert nature [110].

Zirconia, a bioinert metal oxide ceramic, is commonly used for hip and dental prostheses. When mixed with oxides such as MgO and CaO, zirconia gains increased molecular stability. Notably, 3% mol yttria (3Y-TZP) is a widely used additive that produces tetragonal zirconia polycrystal (TZP) [111]. Innovations in material jetting systems for 3D-printed implants have led to faster production of ceramics with properties within the TZP range [112]. Studies comparing AM and subtractive manufacturing methods for 3Y-TZP and alumina-toughened zirconia revealed slight differences in the ceramics' physical properties [111].

Bioinert ceramics offer high mechanical strength and low wear, ideal for permanent scaffold constructs in scenarios where natural bone repair is not feasible. However, the potential for stress shielding necessitates scaffolds that closely mimic the surrounding bone tissue's mechanical properties.

#### Polymers

Polymers, notably biodegradable ones, find broad application in bone, dental fixation, and tissue engineering. These scaffolds must be biocompatible to avoid causing autoimmune reactions or biological damage. Polymers are split into synthetic and natural categories, with more comprehensive reviews available [113].

Synthetic polymers frequently used in 3D printing include PLA, poly(glycolic acid), PLGA, polyurethane, and PCL [113,114]. Natural polymers, divided into protein and polysaccharides, aid cell adhesion and function [115]. Collagen, gelatin, silk fibroin, chitosan, alginate, and hyaluronic acid are commonly used natural polymers.

Despite limited clinical trials for orthopedic applications, in vitro and animal studies show potential. One trial used a PCL-based scaffold for preserving alveolar ridge height after tooth extraction, enabling bone healing and better ridge height maintenance [116]. Another study compared PMMA usage for overdentures, with 3D-printed versions showing better patient outcomes [117].

The clinical usage of 3D-printed soft polymers is limited in orthopedics, likely due to their mechanical strengths. The potential applications of 3D-printed polymer scaffolds are confined to non-load-bearing and minor defects. Their main drawback is the mechanical strength, inferior to bone tissues. Synthetic polymers could work well for non-load-bearing defects, like cranial defects. Natural polymers, being part of the ECM of bone tissue, could repair small load-bearing defects.

#### Metals

1. Bioinert metals

In the realm of bone scaffolding, metallic implants, predominantly fashioned using AM technologies, offer enduring and robust solutions. Metals, especially stainless steel, cobalt chromium, and titanium alloys, form the basis of approximately 70% of such implants, owing to their robust mechanical strength and fracture resistance [118].

Major titanium-based alloys, namely Ti-6Al-4V and Ti-6Al-7Nb, provide high durability and mechanical strength. However, these alloys have a major drawback: the release of toxins due to corrosion, leading to downstream immune responses and chronic inflammation [119]. To mitigate these issues, alloys of Mo, Nb, Ta, and Zr have gained interest, owing to their reduced corrosion and stress shielding effects, thereby offering higher biocompatibility [120].

The bioinert nature of solid metal implants often necessitates bioactive coatings to enhance their integration with surrounding tissue. HAP is a commonly employed coating that aids bone tissue regeneration and osteointegration [121]. Recent AM advancements permit researchers to control the porosity in metals such as Ti and NiTi, potentially stimulating cell proliferation and attachment [122–124]. For instance, Taniguchi et al. [125], using SLM, evaluated the optimal pore size of Ti and TiO_2_ scaffolds. Their findings suggested that a pore size of 600 μm exhibits optimal compressive strength, osteoconductivity, and osseointegration in vivo rabbit models. Recent years have also seen the emergence of W4-Mg and Fe-Mn alloys in the production of metallic scaffolds.

2. Biodegradable metals

Biodegradable materials, as opposed to permanent metal alloys, are ideal for bone tissue regeneration applications aiming to eventually restore functionality. These materials, through the release of ions, have been known to encourage osteogenic and angiogenic activities. However, when these materials are employed for load-bearing scenarios, their varying degradation rates and mechanical characteristics must be considered. Enhancements with degradation-resistant substances and posttreatments might be necessary to control the degradation rate under such circumstances.

Biodegradable metals offer robust support to bone tissue throughout the healing phase and degrade over time, obviating the need for subsequent surgery [126]. They possess significantly higher stiffness compared to polymer-based scaffolds, making them more suitable for load-bearing applications in surgical areas [125].

Common biodegradable metals used in scaffolds include Zn, Fe, and Mg, each exhibiting different degradation speeds. Notably, Mg alloys have been engineered to create biocompatible, degradable, and open-pore metal scaffolds that promote bone formation [127]. However, porous magnesium alloys have increased surface area, leading to accelerated degradation. Experiments using Mg with 4-wt.% Y exhibited slower degradation than pure magnesium scaffolds while retaining biocompatibility [128]. An in vivo study compared 3D-printed scaffolds made of Ti, Mg, and Zn, further highlighting the potential of these materials [129].

#### Composites

Composites like polymer/metals, ceramics/polymer, and metals/ceramic offer a variety of material characteristics such as mechanical strength, bioactivity, and degradation features. For instance, calcium phosphate/polyester composites show promising osteogenic differentiation and cell proliferation [127]. Furthermore, PCL combined with CS and pearl powder stimulates bone regeneration in rabbits [130].

Polymer/metal composites provide higher structural stability due to the metal component, as illustrated by grafted titanium/polymer composites that result in enhanced cell proliferation and adhesion [131,132]. Ceramic/metal composites, like β-TCP coated with Mg, offer high bioactivity and cell adhesion [133].

More advanced composites integrate materials with molecular factors like ECM components, inorganic minerals, and growth factors for custom bone regeneration implants [134]. Successful examples include PLA enhanced with carbohydrate particles and PEEK incorporated with calcium HAP [135,136]. Hydrogels combined with minerals also exhibit positive osteogenic results [137,138].

Materials like demineralized bone matrices and HAP are being utilized due to their inherent growth factors and osteogenic substances [139]. Composite materials incorporating these elements have shown reduced inflammatory responses and improved fusion results [140,141]. Implants printed with PLA and nHAP can be mechanically tuned, showing great customization potential [142]. Thus, traditional ceramics' wide application range in various clinical scenarios is due to their diverse material properties derived from these mixtures.

#### Cell-involved bioprinting

3D bioprinting, an exciting advancement in custom bone implant fabrication, integrates living cells directly into the scaffolds during the printing process (Fig. 3). This approach offers advantages over traditional 3D printing methods, which can struggle with evenly distributing cells postprinting [143]. When designing a scaffold with bioink, it is vital to consider the structure of the tissue being replaced, the appropriate cell type, and the bioink material to ensure both biocompatibility and optimal printability properties like viscosity and mechanical strength [144].

Typically, 3D bioprinting involves stem cells due to their ability to differentiate into various cell types. Ejection printing of bioink, which includes rat bone marrow cells, nano silicate, gelatin, and alginate, has been used to create scaffolds that replicate bone ECM, encouraging osteogenesis and healing critical bone defects [145]. Other studies have highlighted the role of silicon (Si)/silicate in enhancing stem cell viability in the bioink, with silica and calcium hydroxide nanoparticles releasing Si ions in the hydrogel to maintain MSC stemness [146].

Furthermore, researchers have developed bioinks without nanoparticles, using MSCs along with a mixture of fibrinogen, type A gelatin, hyaluronic acid, and glycerol. Such scaffolds can be remodeled into bone in vivo, displaying early hypertrophic characteristics that promote higher levels of vascularization and bone formation [147]. The type of material used and the incorporation of specific drugs can influence MSC differentiation and migration. For instance, scaffolds printed with nifedipine-loaded ethosome and laponite have shown to promote bone repair by influencing the osteogenic differentiation and migration of the bone marrow cells [148].

For emergency situations, the development of in situ printing bioinks, which allow immediate, on-site tissue repair, is particularly noteworthy. Bioinks combined with bone cement can be manually assembled and 3D-printed directly onto a patient for immediate tissue repair [149].

However, the use of MSCs is currently limited to smaller defects due to inadequate vascularization postimplantation [150]. To address this, researchers have explored 3D printing scaffolds with multiple cell types, including MSCs and endothelial cells, to facilitate vascularization. Different strategies, such as dual-ink printing and interlaced printing, have been used to create prevascularized spaces or to ensure even cell distribution in the scaffold [151–153].

The inclusion of cell spheroids during the printing process can further enhance cell-to-cell interactions [154]. For instance, human-derived stem cells and endothelial cells mixed with mineral oil form cell spheroids due to oil–aqueous interactions. When these spheroids are loaded into decellularized ECM/β-TCP struts, they create a vascularized bone scaffold that exhibits robust angiogenesis and osteogenic behavior.

Scaffolds that incorporate cells present considerable potential for clinical applications because of their bioactive properties and adjustable mechanical properties. In a noteworthy clinical study, a patient's bone marrow stromal cells were used in a 3D-printed scaffold for a cleft alveolus reconstruction, with promising results [155]. However, the use of bioactive components like cells may limit the size of the defect that these scaffolds can heal, making them best suited for repairing small defects. Despite this limitation, ongoing investigations into cell incorporation into bioinks show great promise for creating customized treatment plans for patients.

#### Drug-involved printing

Beyond their fundamental roles in bone reconstruction and regeneration, AM bone scaffolds can be tailored to meet patients' unique needs. For instance, where there is a high risk of infection associated with the implant, the scaffold can be imbued with anti-infective properties to safeguard patients from specific bacteria.

One technique to introduce anti-infective agents is by incorporating them into the materials used for printing (Fig. 3). AM TCP scaffolds laden with vancomycin, for example, allow for control over drug release rates by adjusting the drug loading modes and combining with polymers [156]. Similarly, a PCL scaffold impregnated with the antibiotic rifampicin was created using a deposition printer at 60 °C, which prevented any loss of the antibiotic's antibacterial activity. Such scaffolds effectively inhibit bacteria such as *Escherichia coli* (*E. coli*) and *Staphylococcus aureus* (*S. aureus*) [157].

In addition to antibiotics, metal ions have been deployed as anti-infective agents. PCL scaffolds integrated with bioactive glass and gallium (Ga) demonstrated antibacterial activity against methicillin-resistant *S. aureus* and *E. coli* [158]. Similarly, silver nanoparticles were produced in a PCL solution through an in situ reduction reaction and then extruded into PCL/silver nanoparticle filaments for scaffold printing [159]. Another method of introducing anti-infective agents involves grafting them onto printed scaffolds. For instance, printed PLGA/HAP scaffolds were covalently grafted with hydroxypropyltrimethyl ammonium chloride chitosan, which demonstrated both anti-infective and bone regeneration capabilities in infected bone defect models [33]. In some instances, polymer scaffolds were submerged in an antibiotic suspension to acquire anti-infective properties [160,161].

Patients suffering from osteoporosis are more susceptible to bone fractures and defects. In such cases, treatment would necessitate both the use of bone scaffolds and a separate regimen for managing osteoporosis. Drugs such as alendronate, zoledronic, and icariin, all targeted for osteoporosis, have been incorporated into AM bone scaffolds made from various materials including PCL, Ti6Al4V, and calcium phosphate [162–165]. One particular study used freeze-dried platelet-rich plasma to coat AM Ti6Al4V porous scaffolds to boost osseointegration in an osteoporosis animal model [166].

Post bone-related tumor resection surgery, patients require bone regeneration as well as suppression of any remaining tumor cells. In this context, antitumor drugs such as soy isoflavones and doxorubicin hydrochloride have been loaded onto scaffolds printed with TCP and PLGA/TCP [167,168]. These scaffolds enhance bone regeneration while suppressing tumor activity/recurrence. If the drug is loaded during the printing process, a cryogenic environment might be necessary to maintain drug activity [168]. The photothermal performance of metal ions has also been used to combat tumors [169]. Elements like Cu, Fe, Mn, and Co were incorporated into bioactive glass-ceramic scaffolds; in vitro results showed that these scaffolds effectively eliminated tumor cells by inducing hyperthermia, substantially inhibiting tumor growth in vivo.

Apart from these scenarios, the needs of patients with other primary diseases or special conditions can be evaluated by physicians, and patient-specific scaffold customization might enhance clinical outcomes.

### Topography design for customized AM bone scaffolds

When the scaffold material has been selected, the topography design of AM bone scaffold needs to be determined. While bone defect imaging can guide the geography and dimensions of the external contour of scaffolds, the macro/microstructure will influence tissue infiltration, as well as the ingrowth of blood vessels and nerves [170].

#### General influence of parameters in the topography design

Several main parameters in the topography, including porosity, pore size, and pore architecture, have been extensively studied and reviewed [171–175]. The influence of each parameter is summarized in Table. However, when clinicians or engineers customize the topography design for a patient, the target implantation site is the primary factory to take into consideration. This review discusses the topography design for trabecular and cortical bones, 2 major bone classifications.

**Table.**
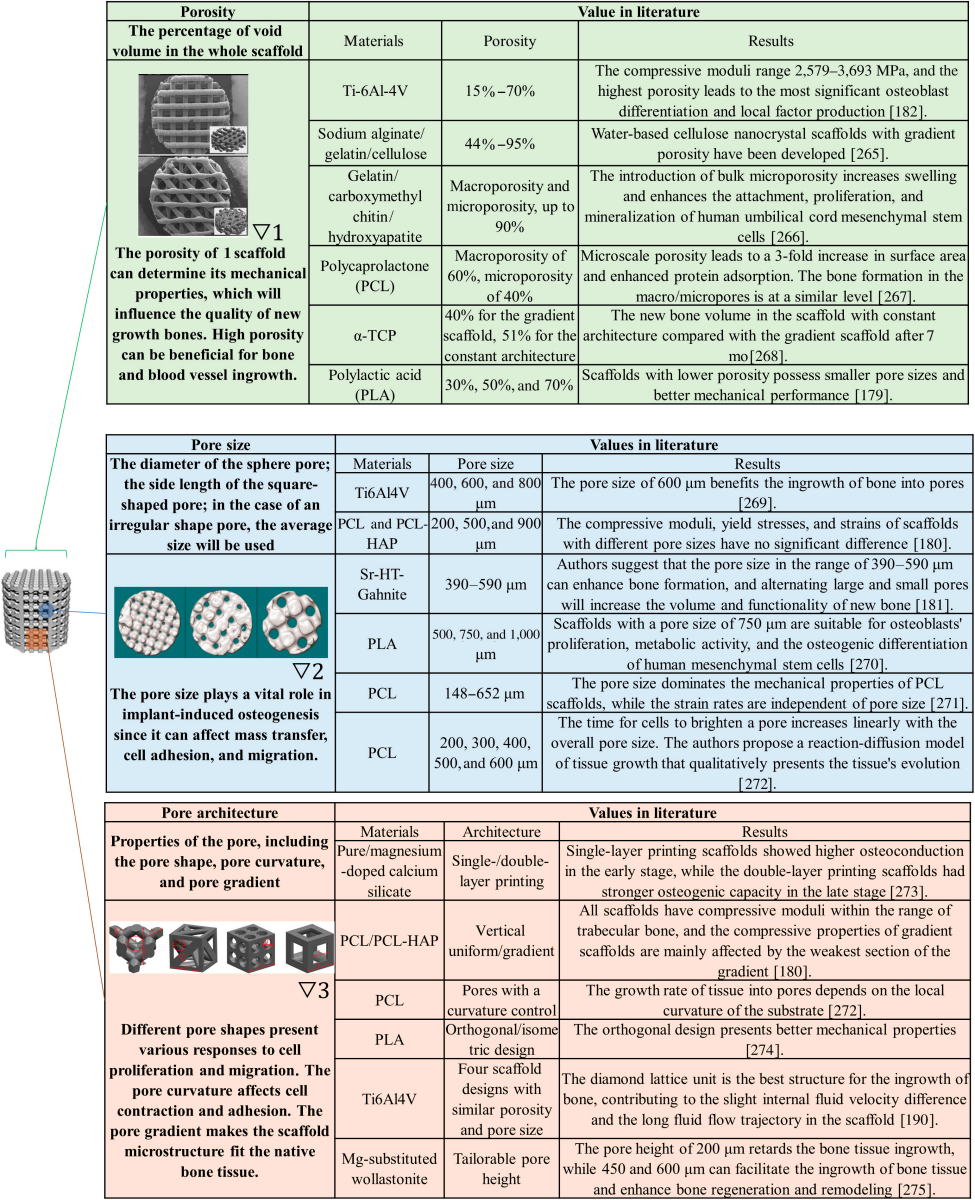
Parameters in the topography design. Pictures are adapted with permission from refs. ▽1 [190], ▽2 [263], and ▽3 [264].

#### Topography design for the regeneration of trabecular bone

For most bone tissues, the mechanical strength mainly depends on the cortical bone, while the trabecular bone provides interconnected space for red bone marrow. Therefore, when a scaffold aims to regenerate trabecular bone tissue, the priority is to mimic the porous structure of the trabecular bone. The trabecular layer of bone possesses porosity in the range of 50% to 90% and compressive strength from 4 to 12 MPa [33,176]. Most studies for trabecular scaffolds adopt uniform topography design, namely scaffolds with repeating cells and consistent porosity [33,34,177,178]. The porosity of these scaffolds is in the range of 14% to 86%, and their compressive strength is within 10 to 200 MPa, following the rule that higher porosities yield lower compressive strengths. Different architectures have shown their influence on the mechanical properties and the ingrowth of new bone [179–181]. There is no conclusion on the optimal architecture, which would depend on multiple factors in clinical application.

Another way to design trabecular scaffolds is to mimic the microstructure of natural trabecular bone. This is a promising way for the customized scaffold since the patient's natural bone structure can be directly involved in the scaffold design. A human femoral head was used as a template by taking images with micro CT and processing them with Scanco software [182]. The template was superimposed at different times to achieve varying porosities between 15% and 70% with compressive moduli between 2,579 and 3,693 MPa. In vitro tests showed decent viability of osteoblasts on these scaffolds, as well as enhanced differentiation. Another study acquired templates from the distal radius trabecular bone [183]. The influence of 3D printing parameters, including input-image resolution, boundary condition, support material, STL mesh decimation, and repetition parameters on mechanical properties, were thoroughly investigated. These process parameters can control the mechanical properties of scaffolds, meaning that the natural bone microstructure can be reserved for the desired biological response, and the sacrifice of mechanical support from the scaffold is avoided. The natural trabecular structure can be further optimized. The Voronoi tessellation method was applied with computer design software to design porous scaffolds [184]. Micro CT images of the L3 human vertebra were processed into 2-dimensional Voronoi cell structures and 3D isotropic porous models, which can be used for 3D printing. These models possess similar histomorphometric indices of trabecular bones. More importantly, these scaffold models' mechanical and fluid properties can be controlled at the beginning of the Voronoi design process. These features provide great potential for clinical application. The biocompatible polymer, ultra-high molecular weight polyethylene, has also been fabricated into trabecular scaffolds using the Voronoi method [185].

#### Topography design for the regeneration of cortical bone

If the scaffold aims to regenerate cortical bones with no demand for mechanical support, the topography design would be similar to the case of trabecular bones. The porous structure, in general, will also be beneficial for the new bone growth and reconstruction in cortical bone regions. However, the topography needs to be optimized when the scaffold is implanted into areas with high load bearing. The challenge is creating scaffolds that maintain porous structures for better bone regeneration outcomes and provide mechanical strength comparable to cortical bones.

One way to enhance the scaffold stiffness is to lower the pore size-to-beam thickness ratio (PO:BT) [186]. The relation between PO:BT and porosity varies in topography so that one can select the low PO:BT with desired porosity. Meanwhile, the scaffold design containing more vertical beams also increases stiffness [186]. Some novel architectures can improve mechanical performance without sacrificing porosity. Glass-ceramic scaffolds, composed of strontium (Sr)-doped hardystonite grains, clusters of submicron gahnite, and a glass phase with a hexagonal architecture, display a compressive strength of 110 MPa, which is within the range of cortical bones [187]. The hexagonal architecture possesses higher compressive and flexural strength than curved, rectangular, and zigzag designs.

Similar to strategies used for trabecular bone, bone-mimic printing offers a promising avenue for generating bone scaffolds with tailored structures. One paper showcases an innovative method for creating bone-like, radial-gradient scaffolds through an extrusion-based 3D (EB-3D) printing technique. The design, inspired by the Koch snowflake fractal structure, results in scaffolds with superior radial porosity and mechanical properties. This approach overcomes conventional EB-3D printers' limitations in fabricating functionally graded scaffolds [188]. Another study presents the successful fabrication of Haversian bone-mimicking scaffolds utilizing digital laser processing based 3D printing. The structural parameters of the scaffold, affecting its mechanical properties and porosity, can be controlled, leading to scaffolds that effectively mimic the native bone structure. The research underscores the potential of biomimetic strategies for designing structured, functionalized biomaterials, contributing to tissue regeneration prospects [189].

For load-bearing implants, metallic materials are strong candidates. Their inherent advantages, such as sufficient mechanical strength and high fatigue resistance, make the topography design of these scaffolds more flexible. AM bone scaffolds made of nonresorbable metals like tantalum, titanium, and nitinol adopt different designs to enhance osteointegration and osteogenesis [190–192]. The architecture of metallic scaffolds can be customized to optimize specific biological responses while their mechanical strengths are comparable to cortical bone. In the case of bioresorbable metallic scaffolds, like magnesium-based scaffolds, the topography design affects mechanical properties and can also influence their biodegradation behavior [193,194]. Smaller pore sizes are considered more promising due to lower hydrogen evolution and smaller reduction of mechanical strength size [195].

### Material-dependent selection of fabrication methods for customized AM bone scaffolds

**Fig. 4.**
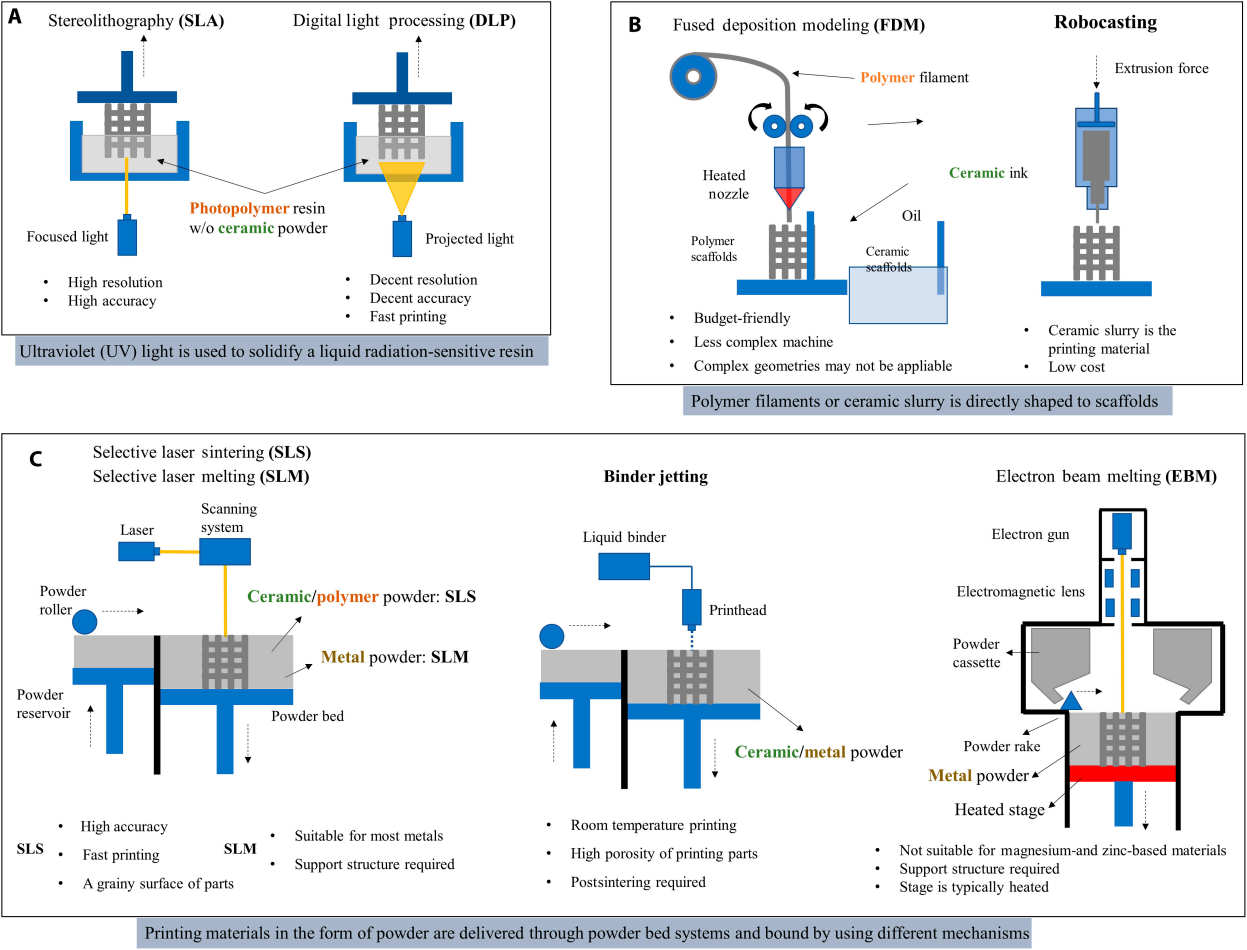
The fabrication methods for customized AM scaffolds. (A) Methods based on UV light-solidification techniques (left: selective laser SLA, right: DLP). (B) Methods based on the direct deposition techniques (left: FDM, right: robocasting). (C) Methods based on the powder bed technique with different powder binding mechanisms (left: SLS/SLM, middle: binder jetting, right: EBM).

Numerous methods exist in AM, many of which are employed to fabricate orthopedic scaffolds. Each fabrication method hinges on unique mechanisms, making them suitable for varied application scenarios (Fig. 4 summarizes these methods and their features). Choosing a fabrication method for AM bone scaffolds involves considering several factors like material type, precision, lead time, surface quality, and postprocessing [196]. In the context of a patient-customized scaffold, the choice of fabrication methods relies heavily on the scaffold material. Hence, this review further discusses AM methods in relation to their performance with different scaffold materials, underscoring the pivotal role of customization.

#### Fabrication methods used for ceramic-based scaffolds

Ceramic-based scaffolds discussed in this review refer to those mainly composed of ceramic materials, which require sintering to strengthen their mechanical stability.

1. SLS

Selective laser sintering (SLS) is particularly advantageous for creating ceramic-based scaffolds in AM. By heating powder particles just below their melting point, SLS enables the formation of solid-state scaffolds from ceramics like HAP and β-TCP [197]. This process, customizable by adjusting parameters such as scanning speed and laser power, can modulate the chemical composition and microstructure of the scaffold [198,199]. By adjusting different parameters such as scanning speed and laser power, designers can customize ceramic scaffolds to meet specific requirements. Furthermore, SLS provides a streamlined fabrication process by eliminating the need for postsintering, underscoring its efficiency in rapid printing of customized ceramic scaffolds.

2. SLA/DLP

Stereolithography (SLA) and DLP methods, both advantageous for creating ceramic scaffolds, function by solidifying a blend of photopolymers and ceramic materials when exposed to light. This is followed by a sintering phase to fuse the ceramics, providing extensive customization opportunities. The trade-off between the two lies in SLA's high resolution but slower speed, while DLP offers faster output but at a lower accuracy.

The versatility of these processes is evident in the diversity of the printing suspension used. This mixture accommodates a variety of ceramic types and other additives, such as dispersing agents and defoamers, thereby enhancing the scaffolds' mechanical and biological properties [200,201]. The ability to dope different components into the ceramic paste during SLA/DLP processing adds another layer of customization to the scaffold's properties [201].

A crucial aspect of SLA/DLP-fabricated ceramic scaffolds is the 2-step sintering process. The initial phase involves lower-temperature sintering to remove photopolymers, and a subsequent higher-temperature phase strengthens the scaffolds' mechanical properties. Careful management of these temperatures ensures the phase of the material is maintained [202,203]. Novel aqueous suspensions have also been developed to address high viscosity and nonenvironmentally friendly factors in traditional suspensions [204].

In conclusion, the SLA/DLP methods provide pronounced advantages for the customization of ceramic scaffold properties due to their versatility in material incorporation, controlled resolution and speed, and phase preservation through tailored sintering processes.

3. Binder jetting

Binder jetting printing, unlike SLA or DLP, fuses ceramic powders directly during the printing process without requiring a polymer-based matrix. A critical preparatory step involves milling the ceramic powders, as particle size impacts the printing and sintering process, ultimately affecting the scaffold's density and strength [205]. Additionally, additives can be included during milling to enhance properties [100].

Various ceramics, including TCP, CS, and HAP, have been successfully fashioned into bone scaffolds using this technique [86,87,100,206,207]. Similar to SLA and DLP, any binder used in the printing process must be removed. However, the method varies; for instance, TCP scaffolds require curing and sintering at specific temperatures [86,100], while HAP scaffolds using a water-based binder only need a drying process [207].

One study has further optimized the binder jetting process for ceramic scaffolds by assessing parameters like layer thickness, build orientation, and binder saturation. Among these, build orientation proved most important [208]. Overall, binder jetting offers an effective and flexible method for customizing ceramic scaffolds.

4. Robocasting

The technique of robocasting offers an alternative strategy for creating ceramic scaffolds. It involves the upfront creation of a specially prepared printing ink that possesses an ideal viscosity to flow through the nozzle, along with a strong shape retention capacity upon deposition. This challenge hinges on the specific composition of the ceramic printing material, and modifications may be necessary if the composition changes or additional additives are required. Various formulations have been developed, including the mix of HAP/β-TCP with a Pluronic F-127 solution, 45S5 bioactive glass with carboxymethyl cellulose water, and a blend of zirconia/alumina powder with a water-based PF127 solution [209–213]. To ensure consistent drying, robocasting typically takes place within an oil bath. Once a self-supporting scaffold is successfully deposited, it undergoes sintering and any additional postprocessing steps.

#### Fabrication methods used for polymer-based scaffolds

1. SLA/DLP

Creating polymer-based scaffolds using SLA/DLP presents challenges, key among them being the need for a photo-crosslinkable primary polymer that is biocompatible or biodegradable. As photopolymers need to be retained in the final product, unlike in ceramic scaffolds, this compatibility is critical. Resins such as poly(trimethylene carbonate) with added HAP particles, commercial polyurethane, and poly(propylene fumarate) of varying molecular masses have been utilized successfully in creating bone scaffolds [214–216]. Polyethylene glycol diacrylate mixed with decellularized tendon ECM has also been deployed as a bioink for scaffold creation via DLP [217]. The main challenge in SLA/DLP lies in formulating the appropriate photopolymer resin; however, once established, the printing procedure aligns with standard SLA/DLP methods.

2. SLS

While SLS can melt most polymers for printing, factors like dimensional inaccuracy due to excess powder bonding, insufficient density from incomplete melting, and poor layer bonding can lead to defects in the polymer scaffold [218]. Hence, the printing parameters substantially impact the scaffold's quality. To enhance bioactivity and mechanical properties, ceramic materials are often integrated into polymer-based SLS scaffolds.

Several polymer–ceramic combinations, such as PCL/HAP [218], PCL–TCP [219], PCL and HAP [220], polylactide and calcium carbonate [221], and PLLA and bioactive glass [222], have been used in SLS printed scaffolds. These studies typically optimize sintering parameters based on the print materials and the polymer/ceramics ratio.

Another research examined the effects of SLS process parameters, including laser power, beam compensation, and laser beam diameter, on the dimensional accuracy and mechanical stiffness of PCL scaffolds [223]. Their findings revealed a strong correlation between the molten cross-section's diameter within scaffold struts, the outer strut diameter, and SLS process parameters.

3. FDM

Fused deposition modeling (FDM) is a widely used method for creating polymer scaffolds, renowned for its ability to control the porosity and structure of scaffolds. The critical challenge lies in formulating specific filaments, particularly those that incorporate ceramics into polymer matrices.

Several combinations of polymers and ceramics, such as PLA with HAP or β-TCP, have been used for bone scaffolds [224]. The resulting mechanical properties and microstructure heavily rely on the filaments' composition, often enhanced with ceramic additives.

Different filaments might demand distinct processing parameters, including extrusion speed, pressure, and temperature [224]. The thermal conductivity of the materials can affect the precision of the structures due to the anisotropic nature of thermal conduction [225]. Also, nozzle diameter is crucial in FDM, with filament diameter needing to match closely for accurate structures [226].

#### Fabrication methods used for metal-based scaffolds

1. SLM / EBM

Utilizing SLM and EBM methods, metal powders are processed using powder bed fusion techniques. These methods create objects by solidifying particles with a laser or electron beam layer by layer. SLM employs a laser source for complete powder melting, while EBM uses an electron beam within a vacuum.

Bio-inert metals, such as stainless steel (SS), titanium (Ti), tantalum (Ta), and cobalt chromium (CoCr), and biodegradable metals like magnesium (Mg), iron (Fe), and zinc (Zn) are typically used for creating metal scaffolds.

Stainless steel, while not a primary choice, has been utilized to fabricate highly porous scaffolds via SLM [227]. More studies focus on pure Ti and Ti-based alloys, due to their properties similar to trabecular bone [228–230]. The Ti-6Al-4V alloy shows variable corrosion resistance depending on whether it is fabricated by SLM or EBM, with EBM presenting better results [228]. The building orientation in the fabrication process has limited impact on osseointegration, but factors such as porosity, heat treatment, and laser manipulations during SLM can affect the scaffold's microstructure and fatigue performance [231–234].

SLM has been used to create Ti-TaNb-Zr alloy and pure Ta scaffolds, while CoCr scaffolds were produced via EBM for bone ingrowth [118,235,236]. Despite challenges related to low melting points and oxidation tendencies, biodegradable metals like Zn and Mg have been processed using SLM. For instance, research on porous Mg scaffolds showed that the biodegradation profile could be adjusted through topological design [193]. Other studies reported successful fabrication of WE43 magnesium alloy and Zn with promising relative density and mechanical properties [129,193,237,238]. Iron and iron-manganese scaffolds, fabricated using SLM, demonstrated differing degradation rates [239].

2. Binder jetting

Binder jetting techniques used in creating metal scaffolds involve binding metal particles on a powder bed and subsequently sintering them.

Fe–30Mn and Fe-Mn-Ca/Mg were processed into scaffolds using such methods. A water-based organic binder was employed, followed by a 2-step posttreatment process. The parts were first cured at low temperatures (200 to 230 °C) for 2 to 3 h to remove the binder and strengthen the structure. This was followed by annealing in a protective gas at a high temperature (1,200 °C). The 3D-printed Fe–30Mn and Fe-Mn-1Ca parts showed open porosities of 36.3% and 52.9%, respectively [240,241].

A study used binder jetting to process Stainless Steel 316 into scaffolds with different lattice geometries [242], following similar printing and sintering parameters as the above Fe-based scaffolds.

Postannealing, all metal parts exhibited shrinkage, with higher shrinkage and lower porosity noted in Fe-Mn samples compared to Fe-Mn-1Ca [241]. Nonetheless, all created scaffolds had satisfactory porosity.

## Future Direction and Challenge

### In situ 3D printing

In situ 3D printing is emerging as a compelling option for creating patient-specific bone scaffolds directly at the site of bone defects. This innovation takes advantage of both the evolution in AM techniques and the broad palette of biomaterials now available, opening new possibilities for precise bone regeneration.

One approach employed a laser-assisted bioprinting system to in situ print a collagen bioink mixed with nHAP. This method was used to treat a calvaria defect in mice, exploring different printing geometries such as disk and ring shapes. The study demonstrated that bone regeneration was influenced by the arrangement of cells within the printed scaffold [243]. In another experiment, an EB-3D printer was used to deploy methacrylate gelatin bioink directly into a long segmental bone defect in a swine model. The printed scaffold was then solidified using an ultraviolet (UV) lamp. Notably, the scaffold was printed and set in place within just 12 min, and after 3 months, notable improvement in bone regeneration was observed [244]. Further, in situ 3D printing was performed with a handheld melt spun printer using a PCL filament doped with zinc oxide nanoparticles and HAP microparticles. The scaffolds produced this way displayed excellent biocompatibility in a subcutaneous model in mice [245].

These examples underline the potential of in situ 3D printing, highlighting its capacity for rapid, site-specific bone scaffold creation, with implications for enhancing patient-specific bone regeneration.

### Smart materials for customized AM scaffolds

Biomaterials have a long history in transforming medicine and can be labeled as “bioinert”, “biocompatible”, and “bioactive” depending on their level of activity in the body. The most recent term for biomaterials “that respond to specific cellular signals” was coined as smart materials [246]. External stimuli like temperature, magnetic fields, light, electric fields, and mechanical stimuli will trigger a change in the material. Customized 3D-printed scaffolds are mostly categorized as bioinert, biocompatible, or bioactive depending on the bioink. These materials do not have the ability to mimic the dynamic nature of tissues and change over time; hence, a term called 4-dimensional (4D) printing is made to categorize customized biomaterials that can autonomously evolve over time or change with external stimuli [247]. 4D printing combines 3D printing and time to create a scaffold that is capable of change. This change will be a result of external energy input such as heat, light, or other environmental stimuli [248]. Various materials that can be used for 4D printing are metal, polymer, proteins, DNA, nanowires, and nanotubes. Aside from 3D printing and 4D printing, various smart materials are already developed manually without 3D printing. These materials are typically piezoelectric and shape changing.

### Deep learning for the scaffold design

The process of designing customized 3D-printed scaffolds can be time-consuming, potentially hindering prompt treatment. Consequently, advanced methods for processing digital medical data for scaffold design are of high importance. Among these, deep learning, a branch of artificial intelligence, shows promising potential. It can predict and generate bone models based on patient data using trained convolutional neural networks [249].

In one study, deep learning was leveraged to reconstruct lower-dose pediatric CT scans, illustrating its potential for handling and processing complex medical imaging data [249]. In another groundbreaking application, 3D deep learning was used to automatically generate cranial implant geometries, hinting at its future utility in creating customized bone scaffolds [250]. Further demonstrating the potential of deep learning, high-resolution trabecular bone microstructures were successfully reconstructed from low-resolution CT scans using a novel method called GAN-CIRCLE [251]. Regarding ink-based printing, the current process of ink development is laborious and inefficient. One study utilized machine learning algorithms, notably the random forest method, to predict and optimize ink formulations, improving accuracy and efficiency in biomedical 3D printing applications [252].

Collectively, these advances highlight how deep learning models could drastically enhance the efficiency of developing custom scaffolds. This, in turn, may lead to a more streamlined and precision-oriented approach to clinical practices involving 3D-printed scaffolds.

### Cooperation model for the clinical application of customized AM bone scaffolds

**Fig. 5.**
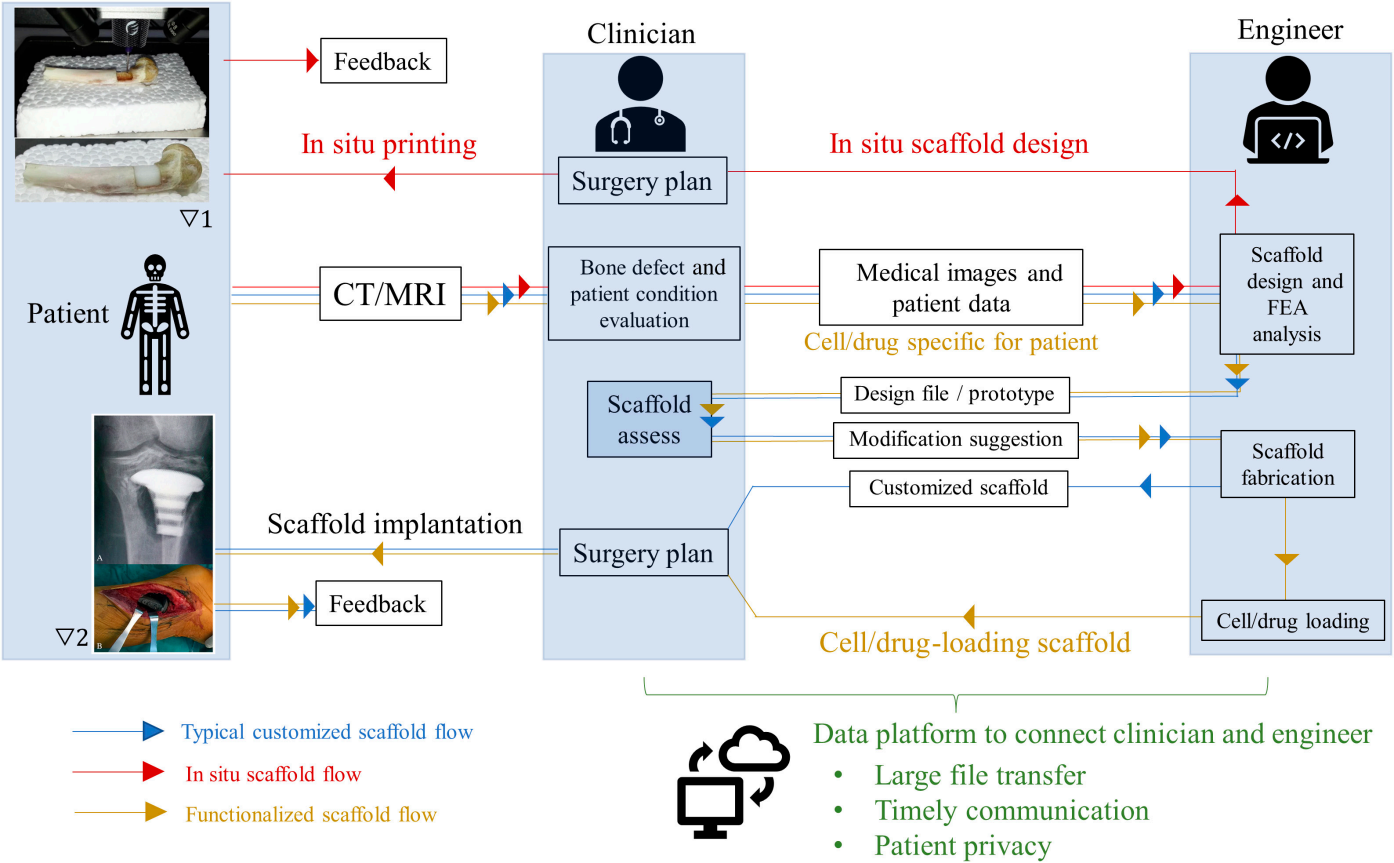
The flow chart shows the process of customizing the AM scaffold for a patient. Pictures are adapted with permission from refs. ▽1 [30] and ▽2 [36].

The clinical deployment of personalized AM scaffolds necessitates a coordinated effort involving the patient, clinician, and engineer. This is a marked departure from the traditional approach, where mass-produced implants are distributed to hospitals. In contrast, AM bone scaffolds are tailor-made, using a design informed by the patient's medical data. Figure 5 elucidates the information flow for an optimal treatment plan utilizing customized AM bone scaffolds. The journey begins with a clinician diagnosing the bone defect, followed by a discussion of treatment options with the patient, and then a consultation with engineers about scaffold possibilities. The clinician acts as an intermediary between the engineer and patient to facilitate the best possible treatment. This model hinges on a close-knit collaboration between the clinician and engineer, both of whom must fully comprehend the customization process at each stage. For cell/drug-loading scaffolds, the clinician collaborates with the patient on cell extraction and supplies patient-specific drugs for scaffold production. Conversely, for in situ printing, the clinician executes the scaffold fabrication guided by the engineer's recommendations.

A critical obstacle in achieving the desired outcome from this cooperative model is the efficiency of communication. Considering the potentially limited or urgent surgical time frame for patients, swift data transmission and interaction between clinicians and engineers are imperative for customizing the scaffold, a process that could be markedly time-intensive. Therefore, a consolidated data platform is vital for timely communication, preserving patient confidentiality, and managing large file transfers, given the breadth of medical data involved.

Another impediment is the complexity of customization. The ideal parameters for bone scaffolds differ for each patient, dependent on their specific condition, necessitating a comprehensive pathological analysis and tailored scaffold design. This extensive workload may result in decreased efficiency and increased costs. However, artificial intelligence, such as the deep-learning technology referenced in Deep learning for the scaffold design, could expedite the analysis and design phases, while quality assessments and evaluations by clinicians and engineers ensure the final outcomes' dependability.

## Conclusion

AM bone scaffolds represent a major advancement in the realm of personalized orthopedic care, offering numerous advantages over traditional and standardized implants. These scaffolds can be designed to precisely fit specific defect shapes and mimic the surrounding host tissue, both mechanically and structurally, based on the treatment strategy and bone defect location. For the successful implementation of precision treatment, customization must be an integral part of all stages. This includes bone defect imaging, scaffold design, and the selection of materials and fabrication methodologies. By adhering to these considerations, we can ensure that the scaffolds produced are tailored to the specific needs of each patient. Ultimately, the proposed cooperative model fosters close collaboration between clinicians and engineers, facilitating the creation of patient-specific AM bone scaffolds. Through this synergistic approach, the true potential of AM in orthopedics can be realized, ushering in a new era of personalized medical treatment.
